# Physiological Mechanisms and Core Genes in Response to Saline-Alkali Stress in Foxtail Millet (*Setaria italica* L.)

**DOI:** 10.3390/biom15060859

**Published:** 2025-06-12

**Authors:** Huimin Wang, Yun Li, Yanan Yang, Yanrui Xu, Xiaoying Fan, Zhenqing Guo, Yucui Han, Xiaohu Lin

**Affiliations:** 1 Hebei Key Laboratory of Crop Stress Biology, College of Agronomy and Biotechnology, Hebei Normal University of Science and Technology, Qinhuangdao 066000, China; cfy@hevttc.edu.cn (H.W.); yyn@hevttc.edu.cn (Y.Y.); mlr@hevttc.edu.cn (Y.X.); fxy@hevttc.edu.cn (X.F.); gzq1035@hevttc.edu.cn (Z.G.); 2 Research Center of Rural Vitalization, Hebei Normal University of Science and Technology, Qinhuangdao 066000, China; ly3974@hevttc.edu.cn

**Keywords:** foxtail millet, saline-alkali stress, cells, physiology, transcriptome

## Abstract

Soil salinization and alkalization are becoming increasingly severe in recent decades, which poses serious threats to crop production and food security in the world. Foxtail millet (*Setaria italica* L.) is an important cereal crop in China, and it is important to elucidate its saline-alkali tolerance mechanisms for the breeding of new saline-alkali tolerant varieties. In this study, we used 75% seawater to treat two foxtail millet varieties with different saline-alkali tolerances (JK3, saline-alkali tolerant; B175, saline-alkali sensitive) during the seedling stage, and conducted morphological, cellular ultrastructure, physiological, and transcriptomic analyses on the two varieties. The morphological analysis of the saline-alkali response indicated that JK3 exhibited stronger saline-alkali tolerance than B175. The results of the cellular ultrastructure showed that under saline-alkali stress, JK3 had a more intact leaf cell structure than B175, indicating that saline-alkali stress causes less damage to its cells. The physiological analysis of saline-alkali response indicated that JK3 had consistently higher activities of catalase and polyphenol oxidase, as well as higher contents of soluble sugars and soluble proteins at 48–120 h than B175. Transcriptomic analysis revealed that JK3 enhanced its saline-alkali tolerance by positively regulating pathways such as tryptophan/fatty acid metabolism, the MAPK signaling pathway, and peroxisome pathways. Further, WGCNA combining morphological and physiological indicators identified four key modules and five functional pathways (MAPK signaling, glycerolipid metabolism, phosphate and phosphonate metabolism, galactose metabolism, and endoplasmic reticulum protein processing) in response to saline-alkali stress, and identified a total of 24 core genes. Functional annotation indicated that these genes may be involved in the response to saline-alkali stress. These findings lay a foundation for in-depth studies of the molecular mechanisms for saline-alkali tolerance in foxtail millet.

## 1. Introduction

Saline-alkali soil is widely distributed in the world, and causes serious negative impacts on agricultural production and the ecological environment [1,2]. Under saline-alkali soil conditions, the high concentration of salts alters the physicochemical properties of the soil and increases the osmotic pressure in the soil solution. This makes it difficult for crop roots to adequately absorb water from the soil, leading to physiological drought and thus posing serious threats to crop yields [3]. According to the survey data of the Food and Agriculture Organization of the United Nations (FAO), more than one billion hectares of lands worldwide are not effectively utilized due to the impacts of salt, and about 60% of them are classified as saline [4]. Currently, China has about 1.48 billion mu of saline soil, accounting for about 10% of the world’s saline land area [5]. The breeding and cultivation of salt-alkali-tolerant varieties are an economically effective method in production practices [6]. Foxtail millet (*Setaria italica*), also known as proso foxtail millet, is a traditional food crop in China, and is characterized by drought resistance, salt-alkali tolerance, and broad adaptability. It is a typical environmentally-friendly crop with an important role in adjusting planting structure, groundwater pressure extraction, and increasing grain production [7,8]. Given that foxtail millet (*Setaria italica*) naturally possesses the characteristic of salt-alkali tolerance, the breeding of salt-alkali-tolerant varieties can significantly increase the yield of foxtail millet in saline-alkali soils, thereby improving the utilization efficiency of saline-alkali lands [9]. Exploring the salt-alkali tolerance mechanism of foxtail millet can provide a crucial genetic basis for the breeding of salt-alkali-tolerant varieties [10].

In recent years, certain progress has been made in research on the mechanisms for the saline-alkali tolerance in plants with interdisciplinary approaches involving molecular biology, genetics, and physiological ecology [11,12,13]. These mechanisms cover several key aspects, including the antioxidant system, osmotic regulation, and ion transport and balance. Firstly, plants can effectively resist saline-alkali stress through their antioxidant systems. Specifically, they have evolved two key defense mechanisms to prevent the excessive accumulation of reactive oxygen species (ROS): one is to enhance the activity of antioxidant enzymes to promote the decomposition of ROS [14], and the other is to synthesize small molecular antioxidants such as glutathione, ascorbic acid, phenolic compounds, flavonoids, and carotenoids for resistance to saline-alkali stress [15]. Secondly, plants can maintain intracellular osmotic pressure balance through osmotic regulation mechanisms to resist saline-alkali stress. This is mainly achieved by the synthesis and accumulation of osmotic regulators such as proline and betaine, which help the plant to maintain normal cellular functions in saline-alkaline environments [16]. Additionally, salt overly sensitive (SOS)-mediated Na^+^ sequestration is also an important pathway for plants to cope with saline-alkali stress [11]. A variety of plants utilize salt-hypersensitive response (SOS)-mediated Na^+^/H^+^ exchangers to sequester Na^+^ in vesicles, thereby reducing the accumulation of harmful Na^+^ and maintaining intracellular ionic homeostasis [17]. Finally, increasing the ratio of potassium (K^+^) to Na^+^ in the cytoplasm is also a key way to enhance Na_+_ tolerance in plants. The entry of Na^+^ disrupts the K^+^ balance, thereby affecting numerous metabolic processes. Therefore, plants can regulate the intracellular content of K^+^ and Na^+^ to maintain normal metabolic activities, which confers the plant with a more effective response to saline-alkali stress [18,19].

Previous research on the saline-alkali tolerance in foxtail millet has been mainly focused on single or double salts, and some studies have found that the components in saline-alkali soil are similar to those in seawater [20]. There are certain differences in the salt-alkali tolerance mechanisms among different germplasm resources within the same species. Jikegu 3 (JK3) is a salt-alkali-tolerant variety bred by our research group. Currently, its salt-alkali tolerance mechanism remains to be explored. A comparison of two genotypes with different salinity tolerance can provide the specific response genes and elucidate the mechanism of salinity-tolerant materials. Therefore, this study utilized seawater to screen for two foxtail millet varieties with different saline-alkali tolerance, explore the saline-alkali tolerance genes in JK3, and analyze the mechanisms for the saline-alkali tolerance of JK3. The findings are of great significance for fully utilizing and developing saline-alkali soil resources and ensuring the sustainable and efficient development of agriculture.

## 2. Materials and Methods

### 2.1. Plant Materials and Salt-Alkali Treatment

This study used the salt-alkali-tolerant foxtail millet variety JK3 and salt-alkali-sensitive foxtail millet variety B175, selected based on morphological analysis by our research group, as experimental materials. JK3 was selected and provided by the Laboratory of Coarse Grain Crops of Hebei Normal University of Science and Technology, and B175 was independently created and provided by Baoding Academy of Agricultural Sciences, Hebei Province, China.

Foxtail millet seeds were disinfected with NaClO and rinsed 3–5 times with water. After the seeds germinated for 24 h, a pot experiment was carried out using vermiculite as the culture medium. (Horticultural-grade expanded vermiculite was selected. Before use, the vermiculite was first soaked and disinfected in a 2% hydrogen peroxide solution for 30 min, and then repeatedly rinsed with deionized water until it became neutral. To ensure the supply of nutrients in the initial stage, the vermiculite was uniformly mixed with 1/2 Hoagland nutrient solution at a ratio of 1:1 for later use.) The conditions were set with a day and night temperature of 28 °C/22 °C, a humidity of 65%, and a day and night duration of 12 h each. After being cultured to three leaves (18D), a 75% concentration of seawater (seawater was sourced from the Bohai Sea area in the Haigang District of Qinhuangdao City, Hebei Province, with a seawater salinity of 2.7 and a pH value of 7.67) was used for saline-alkali stress treatment, and an equal volume of distilled water was used as the control treatment. Each treatment had three biological replicates.

### 2.2. Measurement of Growth Indicators and Physiological Indicators

At 0, 12, 24, 48, 72, and 120 h after treatment, samples of foxtail millet seedlings were taken to determine the growth indicators and physiological indicators. The growth indicators included the plant height (H), fresh weight (FW), dry weight (DW), leaf water content (RWC), and chlorophyll (SPAD value). Indicators of oxidative stress and antioxidant protection included the superoxide anion content (O^2−^), hydrogen peroxide content (H_2_O_2_), malondialdehyde content (MDA), proline content (Pro), soluble sugar content (SS), soluble protein content (SP), superoxide dismutase content (SOD), peroxidase content (POD), catalase content (CAT), and polyphenol oxidase content (PPO). Physiological indicators were measured using a kit method. In addition, the relative electrical conductivity (REC) was measured to assess cell membrane permeability, and the concentrations of Na^+^, K^+^, and Ca^2+^ were measured to understand the effects of saline-alkali stress on ion absorption and distribution in foxtail millet seedlings. Plant height (H) was measured from the base of the plant above ground to the leaf tip using a straightedge. Fresh weight (FW) and dry weight (DW) were measured with an electronic balance. SPAD values were averaged using a portable SPAD-502 chlorophyll meter (Konica Minolta, Tokyo, Japan) at three positions of the second leaf, leaf tip, leaf middle, and leaf root. Leaf water content (RWC) was determined by weighing 0.1 g of the second fresh leaf (FW), soaking it in distilled water for 8 h, and then weighing the saturated fresh weight (TW) when it was at a constant weight, and then drying the absorbed and saturated leaf at 80 °C to a constant weight (DW), and the RWC was calculated according to the formula RWC = (FW − DW)/(TW-DW). Conductivity (REC) was determined by taking 1 g of the second leaf, cutting it up, and placing it in a tube of 50 mL of distilled water for 12 h and shaking it well to determine the initial conductivity, R_1_, and then placing the tube with a stopper into boiling water for 30 min and letting it stand for 20 min at room temperature, shaking it well to determine the conductivity, R_2_. The conductivity of distilled water is recorded as blank conductivity(R_0_). REC was calculated according to the formula REC = (R_1_ − R_0_)/(R_2_ − R_0_). For the determination of ion content, the aboveground tissues were dried at 80 °C until constant, and the samples were weighed, ground, and sieved; the Na^+^ and K^+^ contents (mg-g^−1^) in the plants were determined by H_2_SO_4_-H_2_O_2_ decoction and flame photometry, and the Ca^2+^ content (mg-g^−1^) in the plants was determined by using an inductively-coupled plasma instrument. The content of malondialdehyde (MDA) was determined by the thiobarbituric acid method. The content of O^2−^ was determined by the hydroxylamine oxidation method. The content of H_2_O_2_ was determined by the titanium sulfate colorimetric method. The content of Pro was determined by the sulfosalicylic acid method. The content of SS was determined by the anthrone colorimetric method. The content of SP was determined by the Coomassie brilliant blue method. The activity of SOD was determined by the WST-8 method. The activity of POD was determined by the guaiacol oxidation method. The activity of CAT was determined by the ammonium molybdate method. The activity of PPO was determined by the catechol method. The specific procedures were carried out according to the standards of the reagent kits. The mass of tissue (g) and the volume of extract (mL) were homogenized in an ice bath at a certain ratio, and the supernatant was centrifuged for determination. The kits were purchased from Suzhou Keming Biotechnology Co., Ltd., (Suzhou, China) and the specific methods and formulas were performed according to the instructions. Each treatment was repeated three times.

### 2.3. Observation of the Ultrastructure of Seedling Leaf Cells Under Saline-Alkali Stress

At 72 h of treatment, the control and treated seedling leaves (the second leaf) were taken for ultrastructural observation. Sampled leaves were washed with distilled water; cut into pieces of tissue of about 5 mm along the main veins; quickly immersed in 2.5% glutaraldehyde fixative; left at room temperature for 2 h; rinsed three times with pH 6.8 phosphate buffer for 15 min each time; post-fixed with 1% osmiic acid for 3–3.5 h; dehydrated with ethanol solutions of different concentrations (50%, 70%, 80%, 90%, and 100%) and pure acetone for 15–20 min each time; and replaced with a mixture of ethanol and acetone 1:1. The samples were post-fixed with 1% osmium for 3–3.5 h; dehydrated with different concentrations of ethanol (50%, 70%, 80%, 90%, and 100%) for 15–20 min each time; replaced with a 1:1 mixture of ethanol/acetone and infiltrated with pure acetone for 10 min each; embedded in epoxy resin for 5 consecutive days; and then polymerized in a constant-temperature incubator. Finally, the samples were ultra thinly sectioned, stained by using dibasic uranyl acetate and lead citrate, and placed in a transmission electron microscope for observation [21].

### 2.4. RNA Extraction, Transcriptome Sequencing, and Data Analysis

At 0, 12, and 24 h after treatment, the second and third leaves of foxtail millet were collected and sampled with three replicates, immediately frozen in liquid nitrogen, and stored in a –80 °C refrigerator for RNA extraction, resulting in a total of 18 samples labeled as J0, J12, J24, B0, B12, and B24. After RNA extraction and evaluation by the TRIzol method, a transcriptome library was constructed and sequenced by Shanghai Ouyi Biotechnology Co., Shanghai, China, (NCBI SRA accession number GSE278652). The sequencing data were analyzed using DESeq2 [22] software to screen for differentially expressed genes (DEGs) with fold change ≥ 2 and q-value ≤ 0.05. Clustering and GO [23], KEGG [24] enrichment analyses were performed using R (version number 4.2.2) software to identify significant functional entries.

### 2.5. Weighted Gene Co-Expression Network Analysis

The gene expression data of 18 samples were uploaded to the Ouyi Cloud platform. Low-variability genes (standard deviation ≤ 0.05) were filtered out, and a weighted co-expression network was constructed. Appropriate adjacency matrix weight parameters were selected to meet the scale-free network distribution. Pearson correlation analysis was carried out to identify key modules related to saline-alkali tolerance. GO and KEGG enrichment analysis were performed on DEGs (differentially expressed genes) in the key modules to reveal their functional characteristics. Highly connected hub genes in the key modules were marked with red circles as core genes. The co-expression network was visualized using Cytoscape (version number 3.10.3).

### 2.6. Quantitative PCR Analysis

The cDNA from leaves of the 18 samples sequenced by transcriptome was used as a template. Primers (Table 1) were designed by Primer6 and synthesized by Shenggong Biological (Shanghai) Engineering Co., Ltd., Shanghai, China. A real-time fluorescence quantitative PCR assay was performed using the SYBR GreenⅠ fluorescence quantitative assay kit (Takara, Shanghai, China). The reaction system included 0.3 μL of upstream and downstream primers, 0.75 μL of cDNA (obtained by 2:3 dilution of cDNA and ddH2O), 6.15 μL of ddH2O, 7.5 μL of qPCR SYBR Green Master Mix, and 15 μL of the total system. PCR reaction conditions were as 95 ° C for 30 s, denaturation at 95 °C for 5 s, and 60 °C for 30 s, for 40 cycles. Gene expression was calculated using the 2^−ΔΔCt^ method in triplicate for each sample using EF-1a gene of foxtail millet as an internal control.

### 2.7. Data Statistical Analysis

Data statistical analysis was conducted using Excel 2010, SPSS 26.0, and DPS 7.5 software. Each trait index was calculated based on the measured and control ratio, and was expressed as mean ± standard deviation (mean ± SD). Two-way ANOVA and the least significant difference (LSD) method were used for significance analysis (*p* < 0.05). Data visualization was completed using Excel, Graph Pad Prism 8.0, TBtools (version number 2.091), and Adobe Illustrator 2020 software.

## 3. Results

### 3.1. Effects of Saline-Alkali Stress on Foxtail Millet Growth and Development

**Figure 1:**
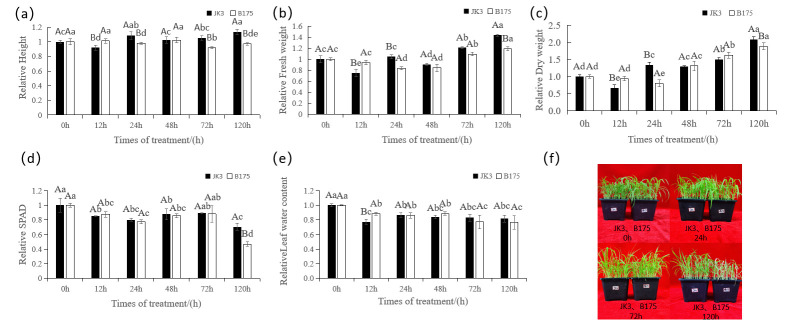
Morphological photographs and growth conditions of JK3 and B175 under control and saline-alkali stress, (**a**) Relative Height. (**b**) Relative Fresh weight. (**c**) Relative Dry weight. (**d**) Relative SPAD content. (**e**) Relative leaf water content. (**f**) Phenotype of JK3 and B175 seedlings under control and saline-alkali stress.Two-way analysis of variance (ANOVA) was performed on all the data. Capital letters indicate significant statistical differences at the *p* < 0.05 level among different varieties at the same treatment time. Lowercase letters indicate significant statistical differences at the *p* < 0.05 level among different treatment times for the same variety. The error bars represent standard deviation (SD). The size of the column indicates the ratio of the treatment to the control.

To explore the effects of saline-alkali stress on foxtail millet growth and development, we analyzed the morphological indicators of foxtail millet seedlings under saline-alkali stress treatment for 0–120 h (Figure 1, Supplementary Table S1). The results revealed that JK3 exhibited higher saline-alkali tolerance than B175. Although saline-alkali stress significantly affected the plant height of JK3 at the early stage, the plant quickly recovered and ultimately showed vigorous growth. In contrast, saline-alkali stress led to an overall decline of B175 plant height (Figure 1a). In terms of fresh/dry weight of the aboveground part, JK3 also demonstrated faster growth recovery than B175 after saline-alkali stress (Figure 1b,c). With the extension of saline-alkali stress, the chlorophyll content in the leaves underwent a “decreasing–increasing–decreasing” change process in both JK3 and B175. At the early stage of stress, the chlorophyll content significantly decreased in both varieties, but a slight rebound was observed at 48 h in JK3. At 120 h after stress treatment, B175 showed a significantly greater decrease in chlorophyll content than JK3, indicating that JK3 has a stronger ability to maintain the chlorophyll content under prolonged saline-alkali stress (Figure 1d). Moreover, JK3 quickly recovered and maintained stable leaf moisture after stress, while B175 showed continuous water loss, ultimately resulting in a lower leaf water content than JK3 (Figure 1e). Overall, JK3 exhibited significantly better growth than B175 under saline-alkali stress, demonstrating stronger growth advantage and adaptability to adverse conditions (Figure 1f).

### 3.2. Effects of Saline-Alkali Stress on the Cellular Ultrastructure of Foxtail Millet Leaves

**Figure 2:**
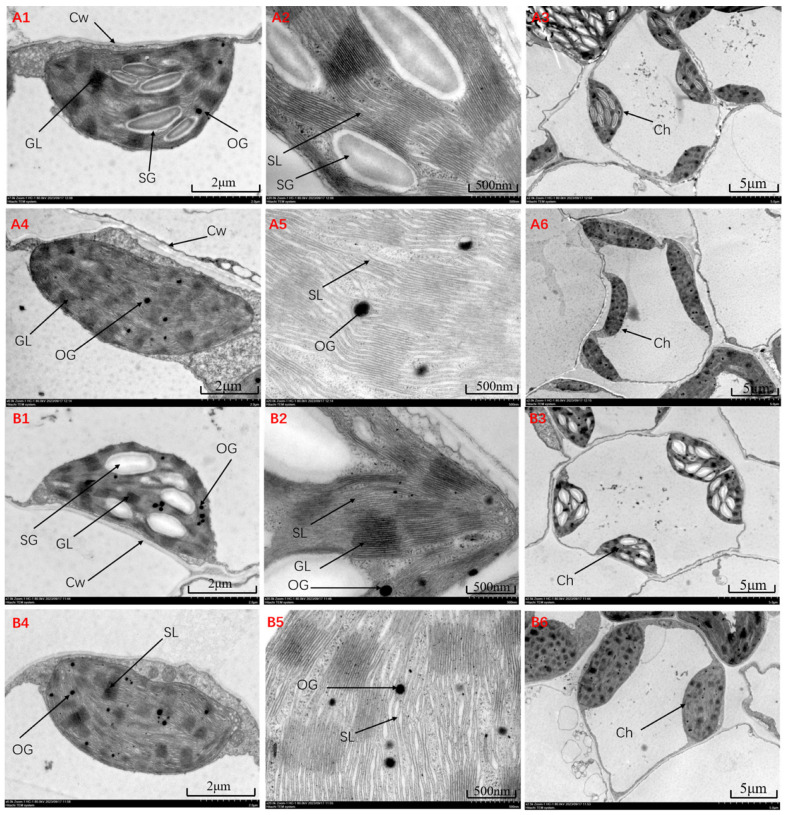
Ultrastructure of leaf cells of JK3 under control (**A1**–**A3**) and saline-alkali stress (**A4**–**A6**). Ultrastructure of leaf cells of B175 under control (**B1**–**B3**) and saline-alkali stress (**B4**–**B6**). GL: grana. SL: stromal layer. SG: Starch granules. OG: osmiophilic granules. Ch: chloroplast. Cw: cell wall.

The observation of the ultrastructure of JK3 and B175 leaf cells (Figure 2) showed that under stress at 0 h, the mesophyll cells of JK3 and B175 were oval-shaped with no plasmolysis, and contained a large number of chloroplasts. The chloroplasts of JK3 were spindle-shaped, while those of B175 were boat-shaped, with intact outer membranes and intact thylakoid structures and well-organized stromal layers in the stroma. Additionally, many starch granules and a small number of osmiophilic granules were evenly distributed within the chloroplasts. At 72 h after saline-alkali treatment, there were some changes in the cell structures of JK3 and B175. The mesophyll cells of JK3 became irregularly oval, with no reduction or dissolution of organelles; the chloroplasts were elongated, and the stacking of stromal layers in the stroma became irregular, with a more dispersed structure and an increase in osmiophilic granules. In B175, the number of organelles decreased or showed certain signs of dissolution, accompanied by the occurrence of plasmolysis. The chloroplasts became elongated oval-shaped, and the thylakoid membranes were ruptured, leading to a loose and twisted internal structure, where no intact stromal layers were observed and osmiophilic granules were significantly increased and enlarged. Therefore, it can be speculated that the stronger saline-alkali tolerance of JK3 is related to the more intact structure of its chloroplasts.

### 3.3. Effects of Saline-Alkali Stress on the Physiological and Biochemical Characteristics of Foxtail Millet Leaves

**Figure 3:**
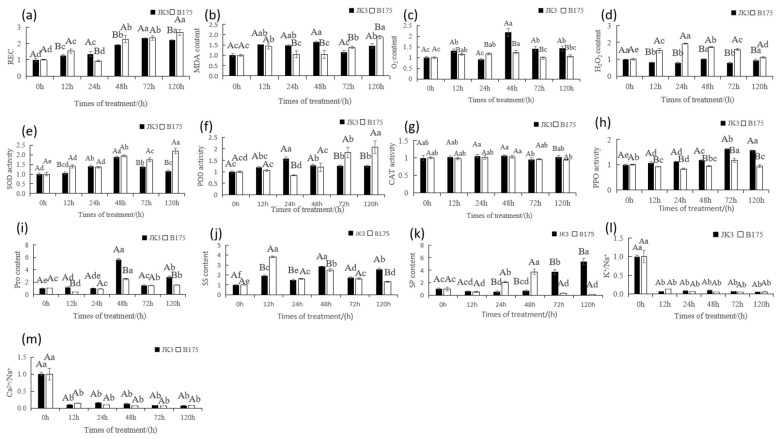
Physiological responses of leaves of JK3 and B175 under control and saline-alkali stress. The physiological indicators (illustrated in subfigures a–m) include: REC (**a**), MDA content (**b**), O_2_^−^ content (**c**), H_2_O_2_ content (**d**), SOD activity (**e**), POD activity (**f**), CAT activity (**g**), PPO activity (**h**), Pro content (**i**), SS content (**j**), SP content (**k**), K^+^/Na^+^ ratio (**l**), and Ca^2+^/Na^+^ ratio (**m**). Capital letters indicate significant statistical differences at the *p* < 0.05 level among different varieties at the same treatment time. Lowercase letters indicate significant statistical differences at the *p* < 0.05 level among different treatment times for the same variety. The error bars represent standard deviation (SD).The size of the column indicates the ratio of the treatment to the control.

To further evaluate the saline-alkali tolerance of the two foxtail millet varieties and understand the physiological mechanisms for their saline-alkali tolerance, we measured the physiological indicators of JK3 and B175 under saline-alkali stress (Figure 3, Supplementary Table S1). Saline-alkali stress significantly increased the REC and MDA content in the leaves of both varieties compared with the control. JK3 has a higher MDA content than B175 at the early stage of saline-alkali stress, but lower MDA and REC contents than B175 at the later stage (120 h) (Figure 3a,b), indicating that B175 experiences more severe membrane lipid peroxidation and greater membrane permeability under saline-alkali stress, while JK3 has stronger and more persistent salt-alkali tolerance to better adapt to saline-alkali stress. In terms of oxidative stress, saline-alkali stress increased the content of H_2_O_2_ and O^2−^ in JK3 and B175 compared with the control, and B175 consistently had significantly higher H_2_O_2_ content but lower O^2−^ content than JK3 (Figure 3c,d). Antioxidant enzyme activity assays showed that under saline-alkali stress, the SOD and POD activity showed a a continuously increasing trend in JK3, while the POD activity showed “increasing–decreasing–increasing” trend in B175; moreover, the enzyme activity in B175 significantly surpassed that of JK3 from 72 h of stress (Figure 3e,f). These results indicated that both foxtail millet varieties are responsive to oxidative stress at the early stage of saline-alkali stress by increasing SOD and POD activity, but higher SOD and POD activity is required in the later stage for B175 to maintain cellular homeostasis. In addition, JK3 always had higher CAT enzyme activity than B175, indicating that JK3 could rapidly decompose the H_2_O_2_ produced by SOD (Figure 3g). The PPO enzyme activity showed continuous significant increases in JK3, with unstable fluctuations in B175, and was 67% higher in JK3 than that in B175 at 120 h (Figure 3h). Osmotic adjustment substances such as proline and soluble sugars significantly increased in both foxtail millet varieties under saline-alkali stress (Figure 3i,j), but JK3 exhibited a higher accumulation of osmotic adjustment substances at the later stage of stress, and the changes in soluble protein content also suggested a stronger adaptability of JK3 to saline-alkali stress (Figure 3k). Furthermore, JK3 could more effectively maintain the K^+^/Na^+^ and Ca^2+^/Na^+^ ratios in the leaves under saline-alkali stress (Figure 31,m), which can prevent an excessive accumulation of Na^+^, thereby enhancing its tolerance to saline-alkali stress. In summary, JK3 demonstrated stronger physiological adaptability and tolerance under saline-alkali stress.

### 3.4. Transcriptomic Analysis of Foxtail Millet Response to Saline-Alkali Stress

In order to explore the DEGs in response to saline-alkali stress in JK3 and B175, we performed RNA sequencing on 18 samples of the two materials under seawater stress at 0 (control), 12, and 24 h, obtaining a total of 125.11 G of clean data. The effective data amount of each sample ranged from 6.86 G to 7.02 G, with a Q30 base distribution ranging from 94.01% to 94.82%, and an average GC content of 53.99%. The alignment rate of the reads obtained from sequencing to the genome was 87.28–98.07% (Supplementary Table S2).

**Figure 4:**
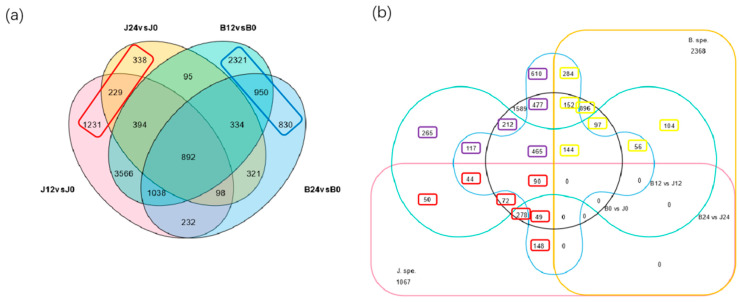
Venn diagram of DEGs among different groups. (**a**) Numbers of DEGs in JK3 and B175 among the comparisons of 12 or 24 h and 0 h (control) of saline-alkali stress. The red and blue rectangles represent the genes that responded specifically to saline-alkali stress in JK3 and B175. The pink, yellow, green, and blue shaded areas represent differentially expressed genes (DEGs) for J12 vs. J0, J24 vs. J0, B12 vs. B0, and B24 vs. B0, respectively. (**b**) Intersection of DEGs among JK3-specific genes, B175-specific genes, B0 vs. J0, B12 vs. J12, and B24 vs. J24. The genes in the red, yellow, and purple small boxes represent the specific genes of subsets 1, 2, and 3, respectively.

To determine the changes in gene expression under saline-alkali stress, we compared the gene expression of JK3 and B175 under saline-alkali stress at 12 and 24 h with that at 0 h to identify the DEGs. In JK3, there were 7680 (J12_vs._J0) and 2701 (J24_vs._J0) DEGs. In B175, there were 9590 (B12_vs._B0) and 4695 (B24_vs._B0) DEGs. The results showed that JK3 had fewer DEGs than B175 under saline-alkali stress. JK3 and B175 contained 1798 and 4101 unique DEGs, respectively (Figure 4a). Among these, 731 unique DEGs in JK3 (Subset 1) showed differential expression between the two varieties, and 1733 unique DEGs in B175 exhibited differential expression between the two varieties (Subset 2). Additionally, among the common DEGs between the two varieties, 2147 showed differential expression between the two varieties (Subset 3) (Figure 4b).

**Figure 5:**
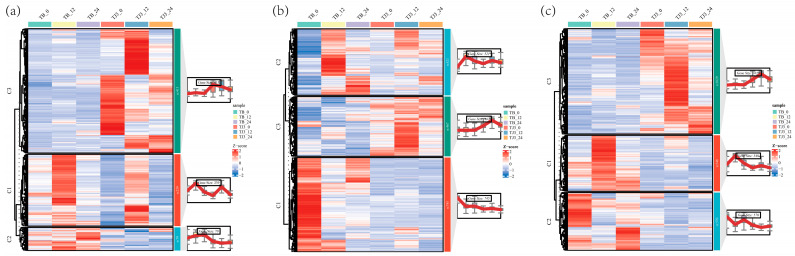
Expression clustering of selected DEGs. Figures (**a**–**c**) show the clustering heatmap of log10 (FPKM+1) values for these DEGs in Subsets 1, 2, and 3, respectively. The box plots show the expression distribution of the associated clusters. Each subset is divided into three clusters, with c1, c2, and c3 representing cluster 1, cluster 2, and cluster 3, respectively.

To explore the potential regulatory genes of tolerance to saline-alkali stress, we performed a co-expression clustering analysis on the aforementioned Subsets 1, 2, and 3, with each subset being divided into three clusters (Figure 5). In Subset 1, 56.50% of the DEGs (413, cluster 3) were highly expressed in JK3. In Subset 2, 73.05% of the DEGs (1266, clusters 1 and 2) were highly expressed in B175. In Subset 3, 1118 DEGs (clusters 1 and 2) were highly expressed in B175, while 1029 DEGs (cluster 3) had high expression in JK3. These results indicated that there are great differences in the regulatory mechanisms of saline-alkali tolerance in the two foxtail millet varieties. In summary, the 1442 DEGs highly expressed in JK3 may positively regulate the saline-alkali tolerance, while the 2384 DEGs highly expressed in B175 may negatively regulate the tolerance.

**Figure 6:**
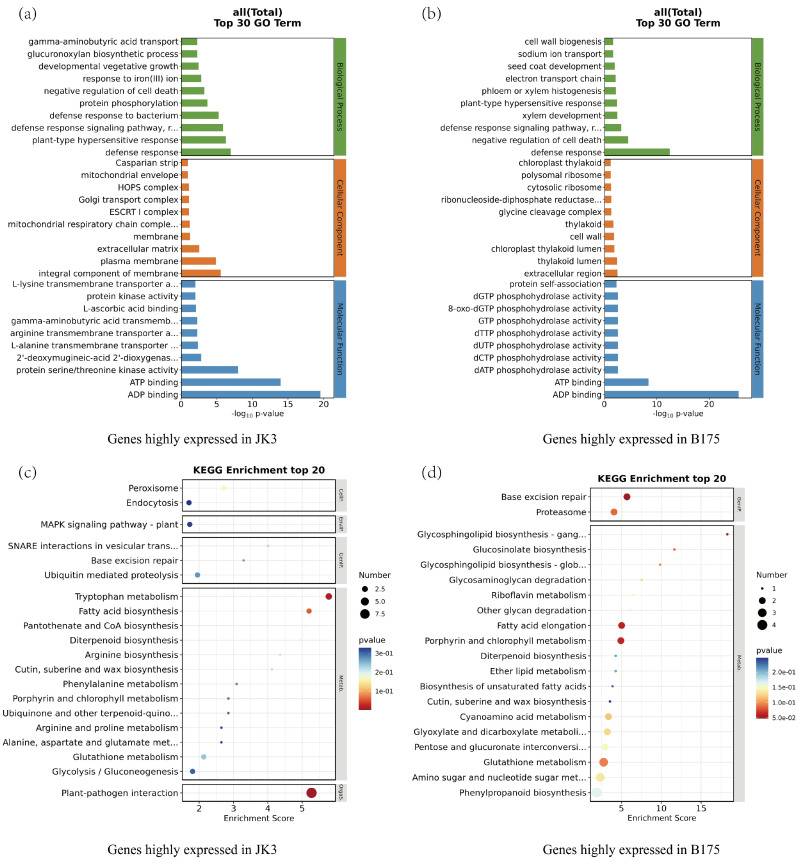
Significantly enriched terms and pathways for the selected DEGs. The top 30 GO pathways are shown for the genes highly expressed in JK3 (**a**) and in B175 (**b**). GO terms were classified into three groups (BP, CC, and MF). The top 20 KEGG pathways are shown for the genes highly expressed in JK3 (**c**) and in B175 (**d**).

To analyze the functions of the 1442 and 2384 DEGs that are highly expressed in JK3 and B175, respectively, we further screened the DEGs that were highly expressed at three time points, namely those in the B0 vs. J0, B12 vs. J12, and B24 vs. J24 comparisons. The results showed that there were 373 and 320 DEGs highly expressed at all the three time points in JK3 and B175, respectively. We then performed GO and KEGG enrichment analyses on these two sets of DEGs. The GO enrichment analysis included three categories, namely biological processes, cellular components, and molecular functions (Figure 6a,b). In biological processes, both sets of DEGs were significantly enriched in defense response, defense response signaling, the negative regulation of cell death, and plant-type hypersensitive response, which are all related to stress response and tolerance. Additionally, the upregulated DEGs were specifically enriched in the biological processes of defense response to bacteria and protein phosphorylation, while the downregulated DEGs were specifically enriched in pathways such as cell wall biogenesis and the electron transport chain. These results indicated that in JK3, signal-related genes are upregulated and activate the signaling processes under stress, while those genes related to the electron transport chain are downregulated. In terms of cellular components, the upregulated DEGs were significantly enriched in integral components of membranes, plasma membranes, and extracellular matrices, indicating that saline-alkali stress upregulates the genes involved in membrane components and extracellular matrices in JK3. In terms of molecular functions, both the upregulated and downregulated DEGs were specifically enriched in molecular functions of ADP binding and ATP binding. Additionally, the upregulated DEGs were also significantly enriched in molecular functions of protein serine/threonine kinase activity, protein kinase activity, and amino acid transmembrane transport. KEGG enrichment analysis showed that the upregulated DEGs were enriched in plant–pathogen interaction, tryptophan metabolism, fatty acid biosynthesis, peroxisome, glycolysis/gluconeogenesis, and MAPK signaling pathways, while the downregulated DEGs were enriched in pathways such as sphingolipid biosynthesis, fatty acid elongation, glucosinolate biosynthesis, proteasome, glyoxylic acid and dicarboxylic acid metabolism, and phenylpropanoid biosynthesis pathways. Furthermore, both upregulated and downregulated DEGs were enriched in pathways related to basal excision repair, porphyrin and chlorophyll metabolism, glutathione metabolism, diterpenoid biosynthesis, and the biosynthesis of cutin, suberin, and wax (Figure 6c,d). The above results revealed differential response pathways of the two foxtail millet varieties to saline-alkali stress.

### 3.5. WGCNA of Foxtail Millet Response to Saline-Alkali Stress

**Figure 7:**
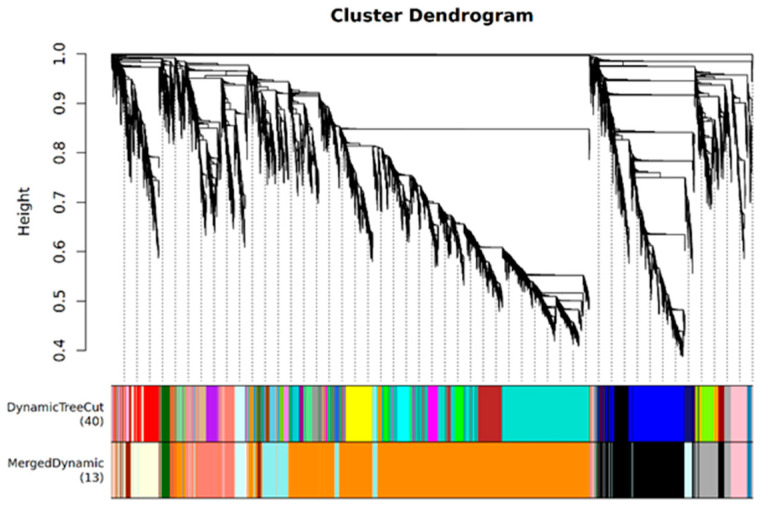
Gene clustering and module recognition in foxtail millet. Different modules are marked with different colors. Each leaf of the cluster tree represents a gene.

**Figure 8:**
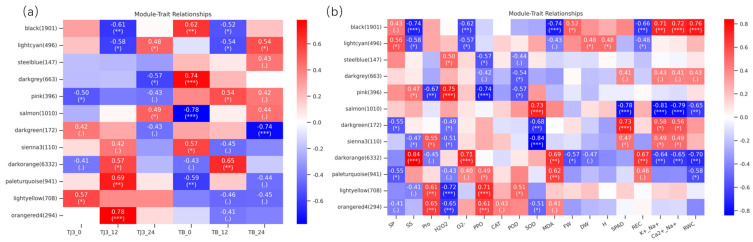
Module–sample/trait correlations and corresponding *p*-values. (**a**) Module–sample correlations and corresponding *p*-values. Each row represents a specific module, and each column represents a sample. The heatmap on the right shows the Pearson correlation between module eigengenes and samples. The numbers in each cell represent the correlation coefficients and correlation significance levels (in parentheses). The color of the cell reflects the degree of correlation. (**b**) Module–trait correlations and corresponding *p*-values. These traits correspond to the eighteen physiological indicators mentioned above. *: Correlation significant at the *p* < 0.05 level. **: Correlation significant at the *p* < 0.01 level. ***: Correlation significant at the *p* < 0.001 level. “.”: The significance level of correlation is between having a certain trend of association and not reaching the significance level represented by “*”.

The above analysis has identified several strategies employed by JK3 to cope with saline-alkali stress, such as enhancing the metabolic level by regulating bacterial defense responses, protein phosphorylation, MAPK signaling, and the expression of stress response-related genes. However, the gene regulatory network of JK3 under saline-alkali stress remains unclear. Therefore, we filtered out the genes with low expression variations (standard deviation) from the 29108 genes obtained from transcriptome analysis, resulting in 13219 genes for WGCNA. A total of 13 co-expression modules were identified (Figure 7). Correlation analysis between the modules and samples showed that the orangered4 module had the closest correlation with J12, and the salmon module had the strongest correlation with J24, indicating that the genes in these two modules are related to the saline-alkali tolerance of JK3. Moreover, the darkorange module had the strongest correlation with B12, and the lightcyan module had the highest correlation with B24, indicating that these two modules are related to the saline-alkali tolerance of B175 (Figure 8a). Further correlation analysis of the modules with morphological and physiological traits revealed that the orangered4 module was significantly positively correlated with Pro and PPO, and significantly negatively correlated with H_2_O_2_ and SOD. The salmon module showed extremely significant positive correlations with SOD and negative correlations with SPAD, K^+^/Na^+^, Ca^2+^/Na^+^, and RWC. The darkorange module had extremely significant positive correlations with SS, O^2−^, MDA, and REC, and extremely significant negative correlations with FW, K^+^/Na^+^, Ca^2+^/Na^+^, and RWC. The lightcyan module had extremely significant positive correlations with SP, DW, and height, but extremely significant negative correlations with SS, O^2−^, and REC (Figure 8b). These results revealed the physiological and transcriptional differences between JK3 and B175 under saline-alkali stress.

**Figure 9:**
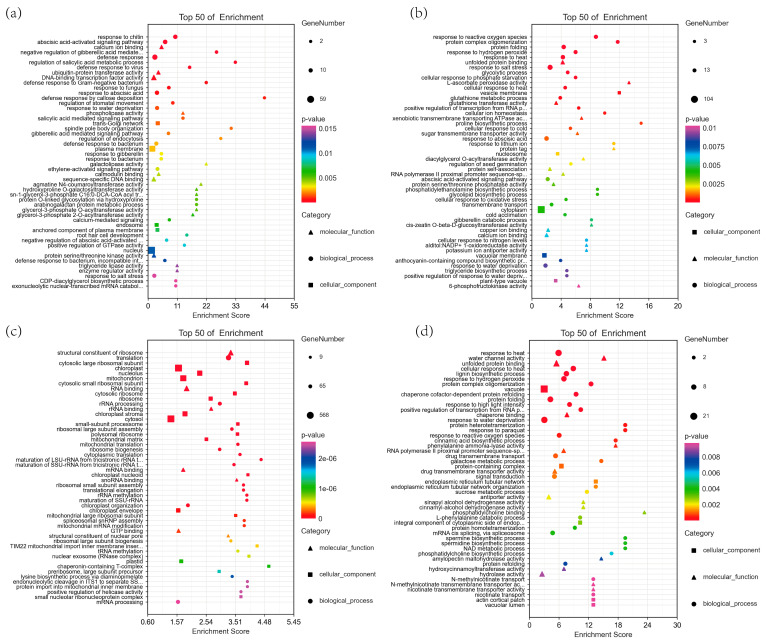
GO pathways of four significant modules. The top 50 GO pathways are shown for the genes from the orangered4 module (**a**), salmon module (**b**), darkorange module (**c**), and lightcyan module (**d**). GO terms were classified into three groups (BP, CC, and MF).

We further conducted a GO enrichment analysis to reveal the functions of DEGs in the four important modules mentioned above (Figure 9). The DEGs in the orangered4 and salmon modules were mainly involved in biological processes related to drought response, salt stress response, abscisic acid (ABA)-activated signaling, and ABA response, which are all associated with plant resistance to abiotic stress. The DEGs in the darkorange module mainly participated in biological processes such as translation, ribosomal RNA processing, and ribosomal large subunit assembly, which play important roles in the response of B175 to saline-alkali stress. The DEGs in the lightcyan module mainly participate in biological processes related to heat response, lignin biosynthesis, and hydrogen peroxide response. To more comprehensively understand the functions of genes in different modules, we also conducted a KEGG pathway enrichment analysis, identifying a total of 39 significantly enriched pathways from the four important modules (Table 2). Among them, the MAPK signaling pathway and glycerolipid metabolism pathway were common to both the orangered4 and salmon modules, while phosphate and phosphate metabolism, galactose metabolism, and endoplasmic reticulum protein processing pathways were shared by the salmon and lightcyan modules, indicating that these five pathways play core roles in the response of foxtail millet to saline-alkali stress. Additionally, “Plant–pathogen interaction” was unique to the orangered4 module, while “Ribosome” and “Ribosome biogenesis in eukaryotes” were unique to the darkorange module. These results suggested that the responses of the two foxtail millet varieties to saline-alkali stress have both common and unique pathways.

**Figure 10:**
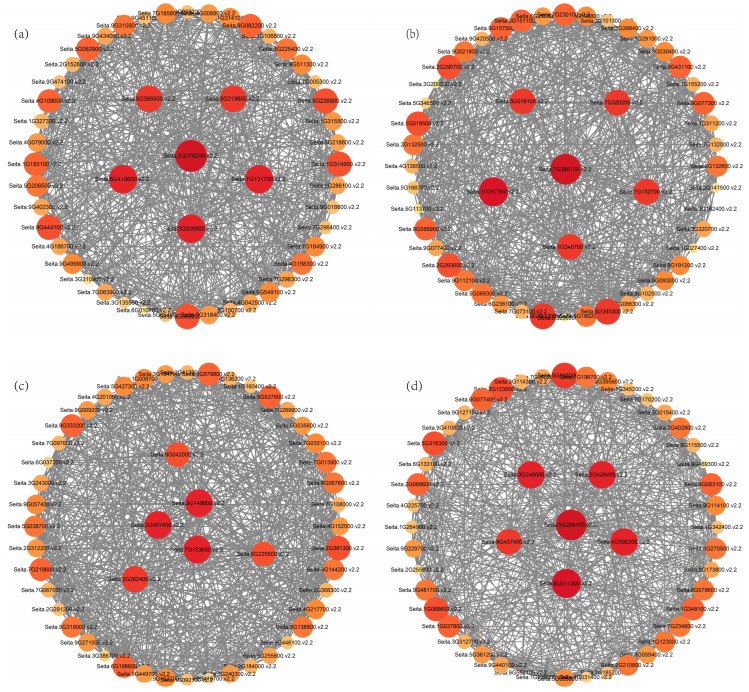
Co-expression network of hub genes in four key modules. The node size indicates the number of interacting genes. Line thickness between nodes reflects the weight value. Figures (**a**–**d**) represent the co-expression networks of hub genes in the orangered4 module, salmon module, darkorange module, and lightcyan module, respectively.

To identify the core genes involved in saline-alkali stress response in foxtail millet, we identified the hub genes in the four key modules, and analyzed and visualized the top 50 connected genes of each module using Cytoscape 3.4.0 (Figure 10). The node size was proportional to the number of interacting genes. The top six genes with the highest connectivity in each module were selected as the hub genes, which may play a core role in foxtail millet tolerance to saline-alkali stress. In the orangered4 module, the six hub genes included two WRKY24 transcription factors (Seita.5G365500.v2.2 and Seita.3G206900.v2.2), one proline-rich receptor-like protein kinase (Seita.5G319600.v2.2), one capsule complex component (Seita.5G419600.v2.2), one disease resistance protein Pik-1 (Seita.7G131700.v2.2), and one gene of unknown function (Seita.5G379200.v2.2) (Figure 10a). In the salmon module, the six hub genes included one ubiquitin fusion degradation protein (Seita.7G280100.v2.2), one amino acid transporter AVTA1 (Seita.1G346700.v2.2), one possible amino acid permease 7 (Seita.7G132700.v2.2), one dehydrin DHN1 (Seita.1G267200.v2.2), one Anamorsin homolog (Seita.3G018100.v2.2), and one gene of unknown function (Seita.7G320200.v2.2) (Figure 10b). In the darkorange module, the six hub genes comprised two exosome complex components RRP411 (Seita.3G149800.v2.2 and Seita.2G401600.v2.2), one mitochondrial GTPase1 protein (Seita.9G042000.v2.2), one DNA topoisomerase 3 (Seita.2G262400.v2.2), one multi-organellar RNA editing factor (Seita.6G225500.v2.2), and one gene of unknown function (Seita.7G153600) (Figure 10c). In the lightcyan module, the six hub genes included one possible E3 ubiquitin ligase gene (Seita.5G298400.v2.2), one F-actin α2 subunit (Seita.2G428400.v2.2), one ATP-dependent zinc metalloprotease FTSH6 (Seita.4G099200.v2.2), one ribulose-1,5-bisphosphate carboxylase/oxygenase activase (Seita.8G251300.v2.2), one serine/arginine-rich splicing factor SR45a (Seita.9G457400.v2.2), and one gene of unknown function (Seita.3G246600.v2.2) (Figure 10d).

### 3.6. qRT-PCR Analysis

**Figure 11:**
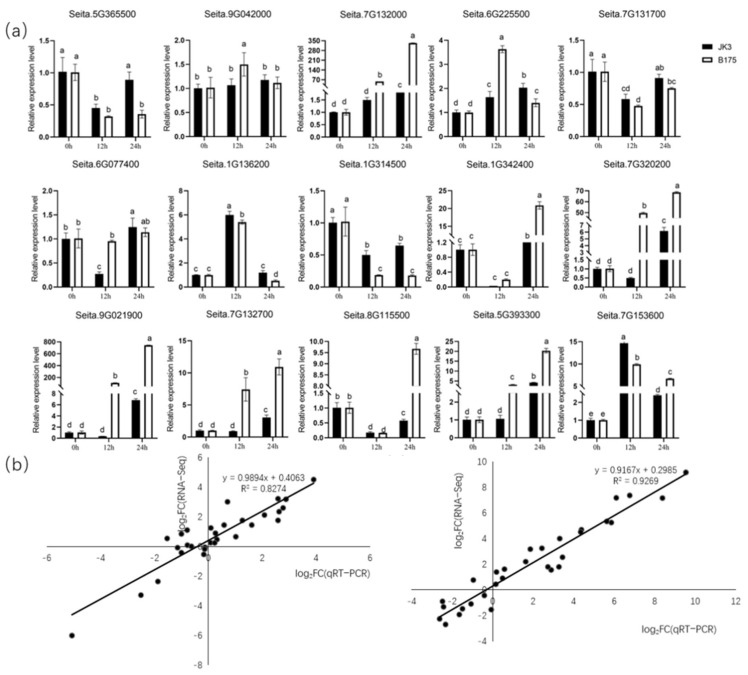
qRT-PCR validation of the transcriptome data. (**a**) qRT-PCR results of 15 genes. (**b**) Validation of gene expression patterns in JK3 (left) and B175 (right). Each point represents a value of fold change in expression level at J12 (B12) or J24 (B24) compared with that at J0 (B0). Different letters indicate significant statistical differences at the *p* < 0.05 level. The error bars represent standard deviation (SD).

To further validate the expression patterns of core genes in the four key modules, we selected 15 genes from 24 core genes for qRT-PCR experiments to determine their expression levels in two foxtail millet varieties at three time points. Ten genes exhibited completely consistent expression patterns, while the remaining five genes showed inconsistent expression patterns across the three time points. Further linear regression analysis showed a significant positive correlation between RNA-seq and qRT-PCR data, indicating that the results of qRT-PCR were generally consistent with the expression patterns of the transcriptome results, validating the accuracy of the transcriptome data (Figure 11).

## 4. Discussion

### 4.1. Effect of Saline-Alkali Stress on Foxtail Millet Morphological Characteristics

The morphological characteristics of plants can directly reflect their responses to external environmental stress and affect their yields to some extent [25]. The soil osmotic potential decreases in a saline-alkali environment, which makes it difficult for plant roots to absorb water, thereby leading to physiological drought, the closure of leaf stomata, and photosynthesis decrease [26,27,28]. Leaves are important photosynthetic organs of plants, and their wilting and curling as well as the loss of greenness under saline-alkali stress directly demonstrate the salt-alkali sensitivity of plants [29]. In this study, the leaves of both foxtail millet varieties showed varying degrees of wilting and loss of greenness under saline-alkali stress, indicating that the plasticity of leaves is an adaptive mechanism for foxtail millet to cope with external stress [30]^.^ The growth of crops is crucial for assessing stress resistance [31]. In this study, the dry/fresh weight of foxtail millet leaves first decreased and then increased under saline-alkali stress, indicating that at the initial stage of stress, foxtail millet is affected by water imbalance and ion toxicity, which inhibit the accumulation of photosynthetic products in the above-ground part. Over time, the root system gradually adapts to the saline-alkali environment, and the plant gradually restores fresh/dry weight through certain regulatory mechanisms, which is consistent with previous research [32]. Furthermore, the RWC of JK3 decreased at 12 h, but gradually recovered and stayed at a stable level at 24 h, while that of B175 continued to decline. This is consistent with the findings of Ouertani et al. [33] that the decrease in leaf water content of salt-tolerant barley genotypes is lower than that of salt-sensitive barley genotypes, indicating that the growth of the saline-alkali-sensitive foxtail millet B175 is more severely inhibited by saline-alkali stress, reflecting the differences in salt-alkali tolerance between the two foxtail millet varieties.

### 4.2. Effect of Saline-Alkali Stress on the Ultrastructure of Foxtail Millet Leaf Cells

Saline-alkali stress can affect plant growth and development, often causing different degrees of damage to cellular structures depending on the plant’s resistance. In plant cells, chloroplasts are the main sites of photosynthesis, and are particularly sensitive to saline-alkali stress, and the integrity and order of chloroplast structure directly influence their photosynthetic capacity [34]. Therefore, under saline-alkali stress, changes in the ultrastructure of chloroplasts can not only reflect the damage caused by the stress but also represent a mechanism by which plants cope with the stressful environment [35]. In this study, saline-alkali stress led to the elongation of chloroplasts in foxtail millet leaves, loose stroma thylakoids, incomplete stromal layers, and increases in osmiophilic granule size and number, which is consistent with previous studies [36,37]. The formation of osmiophilic granules is typically regarded as a sign for the accumulation of thylakoid lipid degradation products, and their changes can intuitively reflect the extent of damage to leaf cells [38]. Generally, a relatively small number of osmiophilic granules are formed in chloroplasts during photosynthesis under normal leaf growth. However, once subjected to adverse stress, the thylakoid structure will be disintegrated, and lipid substances will begin to accumulate, leading to an increase in the number of osmiophilic granules. Therefore, the observed increase in osmiophilic granules in this study is a direct manifestation of the damage to chloroplast membrane integrity. Additionally, JK3 leaves showed relatively intact mesophyll cells with no sign of dissolution; in contrast, B175 leaf cells exhibited a reduction in organelles and signs of dissolution, resulting in plasmolysis and the disorganization of the internal structure of chloroplasts. Previous studies have also indicated significant differences in the extent of damage to mesophyll cells in different salt-tolerant plants under salt stress [39]. These findings suggest that saline-alkali-tolerant foxtail millet experiences less cellular damage under saline-alkali stress compared with saline-alkali-sensitive foxtail millet, which is beneficial for maintaining normal cellular functions under stress.

### 4.3. Physiological Mechanisms of Saline-Alkali Tolerance in Foxtail Millet

The structural stability and functional integrity of biological membranes are closely related to the stress resistance of plants. Saline-alkali stress leads to the substitution of Ca^2+^ on the cell membrane by Na^+^, causing damage to the membrane structure and increasing conductivity [40]. This effect will lead to osmotic stress and ionic stress, resulting in the accumulation of ROS within the plant and the lipid peroxidation of the membranes [41]. The content of the lipid peroxidation product MDA will rise rapidly in a short period, followed by a decrease under the regulation of the peroxidation system. In this study, the conductivity and MDA content significantly increased in the leaf samples of both foxtail millet varieties under saline-alkaline stress, and the increase was more pronounced in B175. Similar results were reported in previous studies, where the relative conductivity and MDA content of both salt-tolerant and salt-sensitive ryegrass increased under salt stress, but the increase was more significant in salt-sensitive ryegrass [42]. These results indicated that saline-alkali stress causes damage to the plasma membranes of foxtail millet seedlings, leading to electrolyte leakage and exacerbating the degree of membrane lipid peroxidation. A longer duration of saline-alkali stress will result in more severe damage to membrane lipids. However, JK3 has a more effective peroxidation regulation system than B175. Antioxidant enzymes within the plant are crucial for scavenging ROS, and provide effective protection on the cell membrane from lipid peroxidation damage. Under saline-alkali stress, although B175 had a relatively low O^2−^ content at the early stage of stress, its H_2_O_2_ content significantly increased with prolonged stress duration, and was notably higher than that of JK3. This result is consistent with the findings of Chu Min et al. [43] regarding the salt tolerance of sorghum, where the salt-sensitive variety consistently had a higher H_2_O_2_ content than the salt-tolerant variety. This phenomenon may be related to the delayed response of antioxidant enzymes (such as SOD and POD) in B175, which failed to effectively prevent the conversion of O^2−^ to H_2_O_2_. In contrast, although JK3 had a higher initial O^2−^ content, it had a more efficient antioxidant system with high CAT enzyme activity, which allows for the rapid decomposition of H_2_O_2_ produced by SOD. Additionally, the continuous increase in PPO content indicated a strong ability to convert O^2−^, which can help effectively control the accumulation of O^2−^ and maintain a lower level of H_2_O_2_. These results align with the findings of Tada Ki et al. [44] in their study of the alkaline stress of willow foxtail millet, where the CAT enzyme activity in tolerant varieties was consistently higher than that in sensitive varieties, and also with the results of Hong Yintian et al. [45] in their study of the salt tolerance of linear chili peppers, where the PPO content increased with the level of stress. These differences indicate various physiological adaptation strategies for plants to respond to salt stress and oxidative stress.

### 4.4. Impact of Saline-Alkali Stress on the Transcriptome of Foxtail Millet

The response of foxtail millet to saline-alkali stress is regulated by multiple pathways, and transcriptome analysis allows a systematic understanding of the saline-alkali tolerance mechanisms of foxtail millet. The transcription levels can vary along with the variations in stress treatment duration or intensity, and different varieties of the same species may exhibit different expression patterns of genes related to saline-alkali tolerance due to genetic differences [46]. There were 229 upregulated and 63 downregulated DEGs in alkali-tolerant rice and 3557 upregulated and 4565 downregulated DEGs in alkali-sensitive rice under alkali stress compared with the control. A total of 567 and 1259 DEGs were found in salt-tolerant and salt-sensitive cabbage compared with the control, respectively, indicating that salt-sensitive varieties have more vigorous responses to salt stress. In this study, the number of DEGs differed between JK3 and B175 under salt stress for 12 h and 24 h, and was larger in B175 than in JK3. This is in agreement with the findings of previous studies that the moderately sensitive varieties of Chinese cabbage [47] and rice [48] have more DEGs, suggesting that salt-sensitive crops are more complex in their transcriptional response to stress. Further analysis revealed that 1442 DEGs unique to JK3 had higher expression levels than those in B175, while 2384 DEGs unique to B175 displayed higher expression levels than those in JK3. It can be speculated that the different ability to coordinate positive and negative regulation between the two varieties may lead to their different saline-alkali tolerance. The enrichment analysis of the 373 DEGs that were unique to JK3 and highly expressed at all three time points revealed their enrichment in pathways related to bacterial defense, protein phosphorylation response, tryptophan metabolism, fatty acid biosynthesis, peroxisomes, glycolysis/gluconeogenesis, and the MAPK signaling pathway. Previous studies have shown that these pathways can participate in and regulate the response to saline-alkali stress [49]. The MAPK signaling pathway can amplify and transmit abiotic stress signals through phosphorylation to participate in biological processes such as plant growth, development, and stress tolerance [50]. MPK genes are key members of the MAPK signaling pathway, and their overexpression enhances the efficiency of the MAPK signaling pathway. It was found that rice transgenic lines overexpressing *OsMPK5* and *OsMPK44* showed higher salt tolerance [51]. Under high salt treatment, the expression levels of wheat *TaMPK4*, TaMPK6, and TaMPK17 gradually increased [52]. In this study, two of the above 373 DEGs (Seita.1G117500.v2.2 and Seita.8G176000.v2.2) were significantly enriched in the MAPK signaling pathway, and their expression was upregulated by 1.41 folds and 3.59 folds, respectively, compared with that of B175, in JK3 at 12 h of stress. This result indicates that the MAPK signaling pathway plays an important role in the response of JK3 to saline stress. The tolerance of plants to saline stress is closely related to the process of fatty acid biosynthesis. ω-3 desaturase can introduce double bonds at specific positions in the fatty acid chain and convert saturated fatty acids or monounsaturated fatty acids into ω-3 polyunsaturated fatty acids, enabling the cell membranes to maintain good fluidity and stability under salt stress. Experiments on transgenic tobacco cells and plants demonstrated that an overexpression of ω-3 desaturase could improve the tolerance of transgenic plants to salt stress [53]. The introduction of sunflower ω-6 desaturase in yeast resulted in enhanced tolerance to NaCl [54], and two of the 373 DEGs mentioned above were significantly enriched in the fatty acid biosynthesis pathway in this study, with the expression of the Seita.9G276700.v2.2 and Seita.9G276800.v2.2 genes being upregulated by 137.6 folds and 66.7 folds in JK3 compared to B175 at 24 h of stress. These results fully indicated that plant tolerance to salinity largely depends on its intrinsic level of fatty acid unsaturation and/or the ability to maintain or regulate fatty acid unsaturation [55].

WGCNA is a method for constructing gene co-expression networks. It uses transcriptome data to identify co-expressed gene groups (modules) and further analyze the relationship between modules and sample phenotypes, thereby efficiently mining the transcriptome data [56]. Zhang Yi et al. [57] and Deng Zhao et al. [58] both identified key modules related to stress using WGCNA. To better identify the key genes related to saline-alkali stress tolerance, we conducted WGCNA using physiological indicators and transcriptome data [59]. Four co-expression modules closely related to foxtail millet saline-alkali stress tolerance were selected, among which the orangered4 and salmon modules were associated with the response of JK3 to saline-alkali stress, while the darkorange and lightcyan modules were related to the response of B175 to saline-alkali stress. Genes in the orangered4 and salmon modules were significantly enriched in pathways related to chitin response and abscisic acid activation signaling. Previous studies have found that these pathways are positively correlated with plant saline-alkali tolerance [60,61]. At the same time, it has been found that genes related to plant hormone signal transduction and protein kinases are easily induced by saline-alkali stress [62,63]. In this study, KEGG enrichment analysis revealed that the MAPK signaling pathway and glycerolipid metabolism pathway were significantly enriched in JK3, indicating their important role in positively regulating the saline-alkali tolerance of foxtail millet. A total of 24 core genes were screened from the four modules, including two WRKY transcription factors and some other genes related to amino acid transport and ubiquitin-protein ligase. WRKY transcription factors, as major components of the abiotic stress response, can specifically bind to *cis*-acting elements in the promoters of downstream genes and regulate the expression of genes depending on this *cis*-element for stress tolerance at specific intensities in a specific time and space, thus enhancing stress tolerance [64]. The expression of both *AtWRKY25* and *AtWRKY33* in *Arabidopsis thaliana* was increased under NaCl treatment, and further studies showed that an overexpression of either *AtWRKY25* or *AtWRKY33* increased salt tolerance in *A. thaliana* plants [65]. The heterologous expression of soybean *GWRKY54* in *A. thaliana* resulted in enhanced salt tolerance in *GmWRKY54* transgenic plants compared to wild-type plants [66]. In this study, the expression of WRKY transcription factors Seita.5G365500.v2.2 and Seita.3G206900.v2.2 was upregulated by 3.7 folds in JK3 compared to B175 in both cases at 12 h of stress. The above results indicate that WRKY transcription factors play a key role in defense against salinity stress.

## 5. Conclusions

In conclusion, JK3 showed stronger salt tolerance than B175 and could maintain a more intact leaf cellular ultrastructure under saline-alkali conditions. JK3 exhibited higher levels of antioxidant enzymes and osmoregulatory substances in the late stage of saline-alkali stress. Transcriptome analysis indicated that JK3 enhanced its saline-alkali tolerance by positively regulating pathways such as tryptophan/fatty acid metabolism, the MAPK signaling pathway, and peroxisome pathways. Finally, 24 core genes related to saline-alkali tolerance were identified.

## Figures and Tables

**Table 1:** Primers for qRT-PCR.

Primer Name	Forward Primer (5′-3′)	Reverse Primer (5′-3′)
Seita.5G365500.v2.2	ACCATCTGCGGCTGCCTCAA	CAGTTGACGCTGGTGCTGATCT
Seita.7G131700.v2.2	GTCGCCTACGTGGTCTTCTCCT	GCGTTGATGCGGTCAGTCTTGA
Seita.1G314500.v2.2	AACGCCGCCGTCAGATACCA	AAGGTCCACTGAGCCGTCACTC
Seita.7G320200.v2.2	TCGCTCTTGTCGCTCTGGTCTC	CTTCTGCCGTCGTGTTGTTGGT
Seita.7G153600.v2.2	TGCATGGCCGGACTGGTTCT	CTCCTCCTGGTCTGCGAGCATT
Seita.9G042000.v2.2	GCGAGAGCCTGCCGATCTGTAT	AGTTCTGCCAACGCTACTTGCC
Seita.6G225500.v2.2	ATCCGTACCCTCCTCCCTCTCA	GGTGCTGCTGGTCGTTGAAGT
Seita.6G077400.v2.2	GCAAGAGCGTCGGCAAGAACA	TTCACCACGGCCACCTTATCCT
Seita.1G342400.v2.2	ACGTGCGGTCTACGGGTGAT	TCCTGATGAAGTTGTCGGCGAA
Seita.8G115500.v2.2	AGTTGTGCGGGTGGGAAGTGT	CGTCGTACTCCTCCAGCAGGTT
Seita.7G132000.v2.2	TCGCAGCCCATCGCATAAGC	TGCTGGTTGCCGCCGTAGAA
Seita.1G136200.v2.2	GCTTTGCGTTCGGTCCTTGAGA	GCAGGTGCATCATCAGCAGGTT
Seita.9G021900.v2.2	ACGTTGCGGTTGTACCTCACTT	AGCTCCACCTCTGGCTTGTTGT
Seita.5G393300.v2.2	TGTGCTCGACTCTGGTGATGGT	TCGGCGGTTGTGGTGAAGGA
Seita.7G132700.v2.2	ACCTCCCAATTCCCAACATGCC	GCCACCTCTGCCGTCTTCTCTA
SiEF-1a	TGACTGTGCTGTCCTCATCA	GTTGCAGCAGCAAATCATCT

**Table 2:** KEGG pathways of the four significant modules.

Module	Pathway ID	Pathway	Number of Genes	*p*-Value
orangered4	ko04626	Plant–pathogen interaction	12	*p* < 0.001
	ko04016	MAPK signaling pathway—plant	9	*p* < 0.001
	ko00561	Glycerolipid metabolism	3	0.036
salmon	ko00480	Glutathione metabolism	20	*p* < 0.001
	ko04141	Protein processing in endoplasmic reticulum	25	*p* < 0.001
	ko00010	Glycolysis/Gluconeogenesis	17	*p* < 0.001
	ko00561	Glycerolipid metabolism	11	0.001
	ko00440	Phosphonate and phosphinate metabolism	3	0.002
	ko00260	Glycine, serine and threonine metabolism	9	0.004
	ko00053	Ascorbate and aldarate metabolism	7	0.006
	ko00620	Pyruvate metabolism	9	0.007
	ko00564	Glycerophospholipid metabolism	11	0.009
	ko00052	Galactose metabolism	7	0.010
	ko04016	MAPK signaling pathway-plant	13	0.012
	ko00051	Fructose and mannose metabolism	7	0.0112
	ko00270	Cysteine and methionine metabolism	10	0.015
	ko00710	Carbon fixation in photosynthetic organisms	7	0.034
	ko00906	Carotenoid biosynthesis	4	0.044
darkorange	ko03010	Ribosome	263	*p* < 0.001
	ko03008	Ribosome biogenesis in eukaryotes	59	*p* < 0.001
	ko03013	RNA transport	86	*p* < 0.001
	ko00290	Valine, leucine, and isoleucine biosynthesis	12	*p* < 0.001
	ko00280	Valine, leucine, and isoleucine degradation	28	*p* < 0.001
	ko00030	Pentose phosphate pathway	30	*p* < 0.001
	ko00230	Purine metabolism	46	*p* < 0.001
	ko00240	Pyrimidine metabolism	33	0.001
	ko00300	Lysine biosynthesis	12	0.001
	ko00340	Histidine metabolism	11	0.002
	ko00220	Arginine biosynthesis	18	0.002
	ko00640	Propanoate metabolism	19	0.003
	ko00261	Monobactam biosynthesis	9	0.005
lightcyan	ko00330	Arginine and proline metabolism	7	*p* < 0.001
	ko04141	Protein processing in endoplasmic reticulum	13	0.001
	ko00940	Phenylpropanoid biosynthesis	14	0.004
	ko00440	Phosphonate and phosphinate metabolism	2	0.013
	ko00052	Galactose metabolism	5	0.015
	ko00500	Starch and sucrose metabolism	9	0.020
	ko00360	Phenylalanine metabolism	4	0.022
	ko00941	Flavonoid biosynthesis	4	0.041

## Data Availability

The datasets generated and analyzed during the current study are available in the Gene Expression Omnibus database (https://www.ncbi.nlm.nih.gov/geo/, accessed on 2 October 2024).

## Supplementary Materials

The following supporting information can be downloaded at https://www.mdpi.com/article/10.3390/biom15060859/s1: Supplementary Table S1: The initial control values of each indicator. Supplementary Table S2: Statistical results of comparison between sample sequencing data and reference genome.
